# Comparison of Natural Head Position in Different Anteroposterior Malocclusions

**Published:** 2013-05

**Authors:** Zohreh Hedayati, Maryam Paknahad, Farbod Zorriasatine

**Affiliations:** 1 Orthodontic Research Center, School of Dentistry, Shiraz University of Medical Sciences, Shiraz, Iran; 2 Student Research Committee, School of Dentistry, Shiraz University of Medical Sciences, Shiraz, Iran; 3 Visiting Fellow, Wolfson School of Mechanical and Manufacturing Engineering, Loughborough University, Leicestershire, United Kingdom

**Keywords:** Orthognathic Surgery; Head; Posture; Cephalometrics, Radiography

## Abstract

**Objective**: The facial esthetics after orthodontic treatment and orthognathic surgery may be affected by the patient’s natural head position. The purpose of this study was to evaluate the natural head position for the three skeletal classes of malocclusion.

**Materials and Methods**: Our sample consisted of 102 lateral cephalometric radiographs of patients aged 15 to 18 years; class I (n=32), class II (n=40) and class III (n=30). Nine landmarks of the craniofacial skeleton and three landmarks of the cervical vertebrae were determined. Variables consisted of two angles for cervical posture (OPT/Hor and CVT/Hor), three angles for craniofacial posture (SN/Ver, PNS-ANS/Ver, and ML/Ver ) and five for craniofacial angulation (SN/OPT, SN/CVT, PNS-ANS/OPT, PNS-ANS/CVT, ML/CVT). The data were analyzed statistically using ANOVA and post hoc tests.

**Results:** PNS-ANS/Ver and SN/Ver differed significantly (p<0.05) among the three groups. There were no significant differences between class I and class II malocclusions for the indicator angles of cranial posture except for ML/Ver. The SN/CVT was significantly different for class I compared to class III patients. A head posture camouflaging the underlying skeletal class III was observed in our population.

**Conclusion:** A more forward head posture was observed in skeletal class III participants compared to skeletal class I and II and that class III patients tended to incline their head more ventral compared to class I participants. These findings may have implications for the amount of jaw movements during surgery particularly in patients with a class III malocclusion.

## Introduction

The use of cranial reference lines to assess anteroposterior skeletal relationships is inherently unreliable. However, they are still widely used for diagnosis and treatment planning in orthodontics and orthognathic surgery. Some basic reference planes such as the sella-nasion (SN) and Frankfort Horizontal (FH) planes vary widely with respect to each other as well as to the true horizontal. Therefore, measurements based on these planes are likely to yield misleading information [1]. As pointed out by Proffit et al. [2], when these measurements are used for orthognathic patients, they may be even more misleading; therefore, the use of the true horizontal or true vertical planes as alternatives seems to be advisable [1,3].

Natural head position (NHP) is a standardized position of the head in the upright posture with the eyes focused on a point in the distance at eye level [4]. This position was used before using any other intracranial reference plane for head positioning. Before invention of the cephalostat, anthropologists used NHP to study skulls [5,6]. The long-term stability of NHP has been demonstrated in a number of investigations. Cooke and Wei [7] found this measurement stable after 3-6 months. Cooke [8] reported stability after five years, and Peng and Cooke [9] documented its stability for as long as 15 years after the initial radiograph. Another important feature of NHP, which makes this parameter important for achieving realistic orthodontic and orthognathic results, is that it represents the individual’s true life appearance. According to previous findings, cervical and head posture are related to different body factors such as stature, ethnicity [7,10,11] gender [10,12], age and facial morphology (mandibular divergence) [13], mandibular size [14] and facial shape [15,16]. In addition, functional factors that influence head posture include nasorespiratory function, temporomandibular dysfunction [17] and bruxism [18]. The relationships between NHP and various malocclusions such as crowding in the maxillary and mandibular dental arches, spacing, overbite, crossbite, midline discrepancies and molar relationships have been studied before [19,20]. 

Solow and Sonneson found a relationship between head position and crowding of 2 mm or more in the anterior teeth. They observed that the craniocervical angle was 3 to 5 degrees larger in this group compared to children without dental crowding [21]. 

Bjork and Marcotte demonstrated that head position was more extended in class II malocclusion, whereas a more flexed head posture was seen in individuals with class III malocclusion [22,23]. A more extended head posture was also reported in a studies conducted by Gonzalez and Manns [24] and Festa et al. [16] that compared children with class II malocclusions and class I occlusions.

Other studies confirmed that head posture changed after different orthognathic surgeries, whereby craniocervical angles (NSL/OPT and NSL/CVT) increased and cervical curvature (OPT/CVT) decreased significantly after mandibular setback surgery [25,26].

The above studies demonstrated the relationship between NHP, different jaw relationships and malocclusions. The facial esthetics after orthodontic treatment and orthognathic surgery may be affected by the patient’s natural head position. 

Determining these relationships appropriately is important in planning for orthodontic/orthognathic treatment. It could be noticed by the amount of jaw movement during surgery to provide a fitting esthetic outcome for patients. A number of studies have compared head and cervical postures for different malocclusion classes in different populations and ethnic origin may influence the head and neck position [10, 11,13 ,19]. The purpose of the current study was to evaluate NHP in a sample of Iranian children with class I, class II and class III malocclusions.

## MATHERIALS AND METHODS

The material for this retrospective study consisted of 102 lateral cephalometric radiographs selected from among 250 radiographs used to compare standard and natural head positions with two techniques for obtaining lateral cephalometric radiographs [27]. All selected radiographs included the first four cervical vertebrae. The remaining 148 with incomplete showing of the fourth cervical vertebra were excluded from the study. 

**Fig1:**
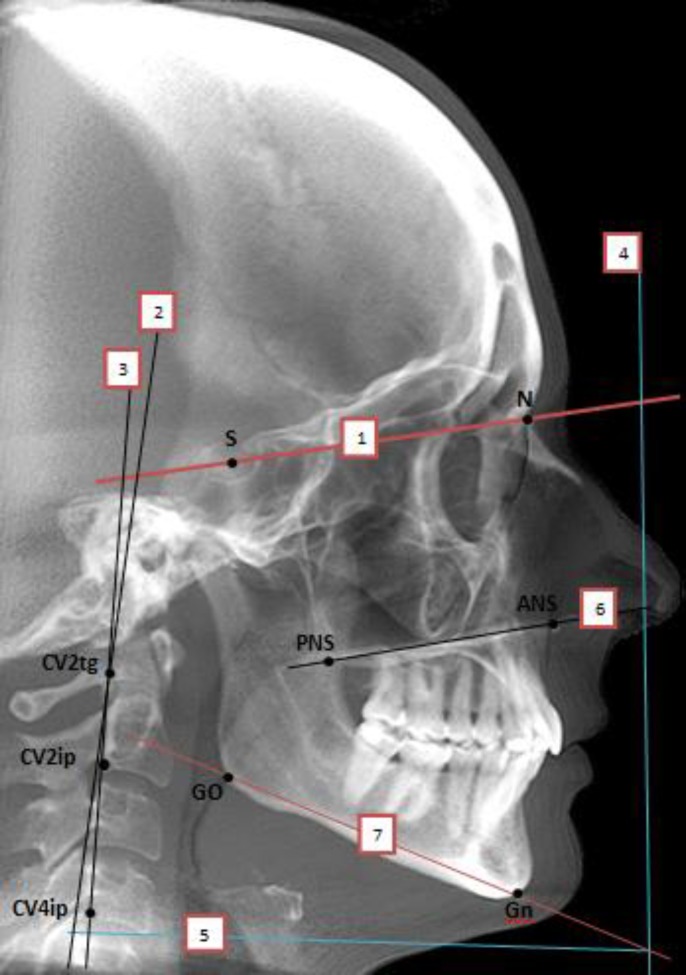
Reference points and reference lines used in this study. Points:S (Sella), N (Nasion),GO (Gonion), Gn (Gnathion), ANS (Anterior Nasal Spine), PNS (Posterior Nasal Spine), CV2tg (Tangent pointof OPT line on the odontoid process of the second cervical vertebra). CV2ip (the most inferior point on the corpus of the second cervical vertebra). CV4ip (The most infero-posterior point on the corpus of the fourth cervical vertebra). Planes: NSL (N-S line), 2) CVT (Cervical Vertebra Tangent). 3) OPT) (Odontoid Process tangent. 4) VER (True Vertical plane). 5) HOR (True Horizontal plane). 6) PNS-ANS. 7) ML ( Mandibular line) ( Go-Gn).

The participants were all from Fars province in southern Iran, and were referred to the Orthodontics Department of The Faculty of Dentistry at Shiraz University of Medical Sciences. The mean age was 17 years ranging from 15 to 19 years. 

They had no history of orthodontic treatment or orthognathic surgery. None of them were

syndromic and all were asymptomatic for temporomandibular joint dysfunction or cervical spine disorders. All participants were screened for nasal obstructions and active symptoms of head, neck, or facial pain. Patients having such problems and also those with severe vertical or horizontal facial growth patterns were excluded from the study. 

Facial growth pattern was determined using GO-Gn/SN (mean=32), FMA (mean= 25) angles and Jarabac index (62-65%). Angles more than the mean and indices lower than the normal range were considered as vertical growth pattern. All of the radiographs were taken in the natural head position using Orthoceph 10E (Siemens AG, Germany). Exposure data were 80-85 kV and 32mA. 

The radiographs were divided into three groups based on their skeletal class: class I (n=32), class II (n=40) and class III (n=30). The proportion of young men and young women in all three groups were equal (16 each for class I, 20 each for class II and 15 each for class III). 

To ensure NHP for the radiographs, a mirror was placed at eye level on the wall in front of the patient, and a plumb line was hung from the film cassette to indicate the true vertical plane (Ver).

Eleven reference points including eight points in the craniofacial area and three points in the cervical column area were marked (Fig 1). These points were marked on the hard copy of each film manually using a sharp pencil. The true vertical (Ver) and true horizontal (Hor) planes were both used in this study (Table 1). 

**Table 1:** Reference Lines Used in This Study

**Cephalometric Reference Lines**	**Description**	**Characterization of Reference Lines**
Hor	True horizontal line	Perpendicular to plumb line
Ver SN	True vertical line	Plumb line
	Anterior Cranial base	Line from sella to nasion
FH	Frankfort horizontal	Horizontal plane from porion to orbital
NA		Line extending from nasion to point A
NB		Line extending from nasion to point B
GoGn	Mandibular plane	Line extending from gonion to gnathion
PNS-ANS	Palatal plane	Line extending from ANS to PNS
**Cervical Region**		
CVT	Cervical vertebra	Posterior tangent to the odontoid process from Cv4ip
OPT	Odontoid process tangent	Posterior tangent to the odontoid process from Cv2ip

**Table 2:** Variables Used in This Study

**Variables**	**Description**	** Characterization of Reference Lines**
SNA	Prognathism of the maxillary apical base to cranial base	Sella-Nasion- A angle
SNB	Prognathism of the mandibular apical base to cranial base	Sella-Nasion- B angle
ANB	Difference between SNA and SNB	Point A-Nasion-point B angle
**Cervical Posture**		
OPT/Hor	Odontoid angle	The angle between OPT line and Horizontal line
CVT/Hor	Upper cervical column posture	The angle between CVT line and Horizontal line
**Craniofacial Posture**		
SN/Ver	Anterior cranial base inclination	Downward angle between SN and Vertical line
PNS-ANS/Ver	Palatal line inclination	Downward angle between PNS-ANS and Vertical line
ML/Ver	Mandibular line inclination	Downward angle between Go-Gn and Vertical line
**Craniofacial Angulation**		
SN/OPT	Craniofacial posture	Downward opening between SN and OPT line
SN/CVT	Craniofacial posture	Downward opening between SN and CVT line
PNS-ANS/OPT	Maxillary base inclination upon cervical column	Downward opening between PNS-ANS and OPT line
PNS-ANS/CVT	Maxillary base inclination upon cervical column	Downward angle between PNS-ANS and CVT line
ML/OPT	Mandibular base inclination upon cervical column	Downward angle between Go-Gn and OPT line
ML/CVT	Mandibular base inclination upon cervical column	Downward angle between Go-Gn and CVT line

The measured variables consisted of two angles for cervical posture (OPT/Hor and CVT/Hor), 

three angles for craniofacial posture (SN/Ver, PNS-ANS/Ver , and ML/Ver ) and five for craniofacial angulations (SN/OPT, SN/CVT, PNS-ANS/OPT, PNS-ANS/CVT, ML/CVT) (Table 2). Skeletal class was determined according to ANB angle (normal range of +2 to +3 degrees) and Wits appraisal (normal range -1 to 0 mm) after clinical examination and profile evaluation. All measurements were made by the same investigator. Intra - observer error was calculated after remeasuring the variables in 30 radiographs (10 randomly chosen from each group) 2 weeks after the initial measurement. The data were analyzed by SPSS v. 11.5. Significance of the differences among the three groups was tested with ANOVA. Post-hoc tests (LSD) were used to compare groups. P values less than 0.05 were considered statistically significant.

## Results

The intra-observer error analysis (kappa statistics) showed no significant differences for any variables in the three data groups (P=0.697). We found statistically significant differences between groups in SN/Ver and PNS -ANS /Ver.

There were no significant differences between class I and class II individuals in indicator angles of cervical posture and craniofacial angulation. The only significant difference was observed in the craniofacial posture index ML/Ver (Table 3).

However, a difference can be observed between indicators of cervical posture between class III patients and the other two groups that was not statistically significant, but can indicate a mild straighter inclination of cervical vertebra in class III patients. 

Table 4 compares data for class I and class III skeletal bases. 

**Table 3:** Variables Compared in Class I and Class II Groups

**Variable**	**Class I** **n=32**				**Class II** **n=40**			**P value**
	**Mean**		**SD**	**SE**	**Mean**	**SD**	**SE**	
**OPT/Hor**		88.53	6.609	1.168	88.40	7.421	1.173	0.932
**CVT/Hor**		83.16	7.432	1.314	84.25	6.894	1.090	0.474
**SN/Ver**		79.19	6.761	1.195	79.20	5.566	0.880	0.993
**PNS-ANS/Ver**		87.09	6.301	1.114	87.40	5.883	0.930	0.826
**ML/Ver**		112.75	5.825	1.030	118.75	16.943	2.679	0.039^*^
**SN/OPT**		103.44	9.211	1.628	102.48	9.165	1.449	0.635
**SN/CVT**		108.72	9.861	1.743	106.73	8.773	1.387	0.339
**PNS-ANS/OPT**		93.94	8.824	1.560	92.75	7.915	1.252	0.508
**PNS-ANS/CVT**		98.59	9.172	1.621	96.23	8.310	1.314	0.215
**ML/OPT**		68.34	8.095	1.431	65.95	9.254	1.463	0.276
**ML/CVT**		73.31	7.394	1.307	70.43	8.797	1.391	0.183

* P <0.05

Both SN/Ver and PNS-ANS/Ver angles were significantly larger in skeletal class III adolescents compared to class I (P<0.05). We found that the average SN/CVT angle in skeletal class I adolescents was 4.99 degrees greater than in individuals from skeletal class III. This was the only craniofacial angle which differed significantly among groups (P=0.027).

Data in Table 5 demonstrate that SN/Ver and PNS - ANS/Ver angles were significantly larger in skeletal class III adolescents compared to the class II (P<0.05). Total cervical posture (CVT/Ver and OPT/Ver) did not differ significantly among the three skeletal classes in term of inclination of the upper (OPT) and middle (CVT) segments of the spinal column. 

The ML/Ver angle did not differ significantly between class I and class III groups.

Moreover, the other two indicator angles of cranial posture were significant between these two group individuals. However, the ML/Ver was the only significant angle of cranial posture between class I and class II.

## Discussion

Despite shortcomings of ANB, it is still used in many studies. The ANB angle is affected by rotations and variations in sagittal and vertical jaw dimensions relative to cranial base. As an alternative the Wits appraisal is among the possible alternatives as a replacement for ANB. 

The findings of such studies underscore the necessity of applying both measurements to accurately estimate the anteroposterior relationship of apical bases [28].

**Table 4:** Variables Compared Between Class I and Class III Individuals

**Variable**	**Class I** **n=32**			**Class III** **n=30**			**P Value**
	**Mean**	**SD**	**SE**	**Mean**	**SD**	**SE**	
**OPT/Hor**	88.53	6.609	1.168	86.10	4.880	0.891	0.145
**CVT/Hor**	83.16	7.432	1.314	82.87	4.167	0.761	0.859
**SN/Ver**	79.19	6.761	1.195	83.13	6.279	1.146	0.013^*^
**PNS-ANS/Ver**	87.09	6.301	1.114	90.50	5.251	0.959	0.024^*^
**ML/Ver**	112.75	5.825	1.030	118.30	8.762	1.6	0.074
**SN/OPT**	103.44	9.211	1.628	100.33	6.630	1.210	0.155
**SN/CVT**	108.72	9.861	1.743	103.73	7.311	1.335	0.027^*^
**PNS-ANS/OPT**	93.94	8.824	1.560	93.57	5.131	0.937	0.847
**PNS-ANS/CVT**	98.34	9.172	1.621	96.73	5.971	1.090	0.362
**ML/OPT**	68.34	8.095	1.431	66.83	10.251	1.873	0.409
**ML/CVT**	73.31	7.394	1.307	70.37	10.890	1.988	0.240

* P <0.05

Some studies showed a significant correlation coefficient between the ANB angle and Wits appraisal, but still r values were relatively low. 

In our study, we used both measurements and clinical examination to assure the accurate relationship of apical bases for classifying them into three groups. Because severe vertical and horizontal growth patterns of the face can affect the accuracy of both measurements, we excluded all radiographs with such a problem. A number of studies have investigated the neck and head posture in different malocclusions. The importance of this posture lies in its effect on facial appearance, a factor that can lead many patients to seek orthodontic or surgical treatment. Changes in the function or morphology in this area (i.e., constructing a new occlusion) may lead to changes in the head posture. Our study was conducted to achieve a better understanding of head posture in a southern Iranian population with different anteroposterior skeletal malocclusions.

Some previous studies included only males or females. D’Attilio et al. [17] investigated cervical posture in females. 

In the current study, we did not distinguish between gender or age subgroups. Gresham and Smithells compared 61 children with a poor neck posture to a control group, and showed that children with 'poor posture' had longer faces and a significant increase in the prevalence of Angle's class II malocclusion [29].

The relationship of head posture with class II malocclusion, which showed that 'upright' posture of the head and greater extension of the spinal column were more evident in individuals with class II malocclusion, was also documented by Arntsen and Sonnesen [30]. 

Similar findings have been reported by Gonzalez and Manns [24] and Festa et al. [16]. 

In the current study; however, no significant difference was observed in head posture between class I and class II individuals.

**Table 5:** Variables Compared in Class II and Class III

**Variable**	**Class II** **n=40**			**Class III** **n=30**			**P-Value**
	**Mean**	**SD**	**SE**	**Mean**	**SD**	**SE**	
**OPT/Hor**	88.40	7.421	1.173	86.10	4.880	0.891	0.147
**CVT/Hor**	84.25	6.894	1.090	82.87	4.167	0.761	0.374
**SN/Ver**	79.20	5.566	0.880	83.13	6.279	1.146	0.010^*^
**PNS-ANS/Ver**	87.40	5.883	0.930	90.50	5.251	0.959	0.030^*^
**ML/Ver**	118.75	16.943	2.679	118.30	8.762	1.6	0.878
**SN/OPT**	102.48	9.165	1.449	100.33	6.630	1.210	0.300
**SN/CVT**	106.73	8.773	1.387	103.73	7.311	1.335	0.160
**PNS-ANS/OPT**	92.75	7.915	1.252	93.57	5.131	0.937	0.655
**PNS-ANS/CVT**	96.23	8.310	1.314	96.73	5.971	1.090	0.793
**ML/OPT**	65.95	9.254	1.463	66.83	10.251	1.873	0.840
**ML/CVT**	70.43	8.797	1.391	70.37	10.890	1.988	0.979

* P <0.05

One of the most important findings in our study was that patients with class III malocclusion bent their head forward more than class I or class II individuals; hence PNS-ANS/Ver and SN/Ver angles were significantly larger in patients with class III malocclusion. This result seems to be associated with the finding that SN/CVT angle in class III individuals was significantly smaller than class I participants, and suggests that class III patients tucked their chin in toward their chest more than participants with normal occlusion. Marcotte [23] and Bjork [22] noticed that individuals with a retrognathic facial profile and an obtuse cranial base angle tend to keep their head more extended and hold their foreheads back with their chins somewhat protruding (dorsal). In contrast, persons with prognathic facial profiles tend to have a more acute cranial base angle and hold their chin inclined toward the chest (ventral). 

Bjork [22] theorized that the relationship between the form of the cranial base and craniofacial morphology was often masked by the posture of the head on the cervical vertebra and concluded that the size and position of the mandible is strongly related to the head posture. Changes in head posture have been reported also after orthognathic mandibular surgery. Muto et al. [25] observed an increase in N-S line to OPT (NSL/OPT) and N-S line to CVT (NSL/CVT) angles in relation to head extension after mandibular setback surgery and change of jaws relationship from class III to class I. 

Therefore, these relationships may be important with regard to the amount of jaw repositioning needed for orthognathic surgery in patients with class III malocclusion. Severity of malocclusion and used surgical techniques and anatomical considerations determine the outcome of orthognathic surgery. Unfortunately these are important factors that limit wanted or expected changes even considering NHP in treatment planning.

The ML/Ver was shown to be significantly different between class I and class II groups.

This angle demonstrated almost similar means when comparing class II and class III. However, the difference existed in standard deviations (SD). SD was greater in class II sample indicating more variation in the mandibular growth pattern in this group of our study. In a study performed by D'Attilio et al. [17], this measurement was non significant between all three groups.

In the current study we found no significant differences among the three skeletal classes in cervical posture. Previous studies also failed to document significant differences in the inclination of the upper (OPT) and middle (CVT) segment of the spinal column and craniofacial morphology. Although AlKofide and AlNamankani [19] reported significant differences in the inclination of the lower segment (EVT) of the spinal column between the three classes, our patients with skeletal class III malocclusion had a slightly, but not significant straighter spinal column in the area between the upper and middle segment of the spinal column than patients in skeletal class I or II. This finding can be considered an effect of the differences in the development of the upper and the middle sections of the spinal column in this group. There were some limitations regarding our investigation. Since we used patients' files, we had no direct access to determine confounders and they had to be ignored. 

The presence of some differences between findings of this study and previous studies suggests the need for further studies in this field in other populations by considering and eliminating confounding factors. 

## CONCLUSION

The findings of this study may be useful in treatment planning orthodontics and orthognathic surgery. Our data show that inclination of the upper and middle areas of the cervical column did not differ significantly between patients with class I, class II or class III occlusion. We noted a more forward head posture in skeletal class III participants compared to skeletal class I and II and that class III patients tended to incline their head in toward the chest (ventral) compared to class I participants. These relationships may be important with regard to the amount of jaw repositioning needed for orthognathic surgery in patients with class III malocclusion.
